# Maltine

**Published:** 1879-12

**Authors:** 


### MALTINE.

SOME months ago we received a liberal supply of samples of Maltine and its various compounds. We have not only used them in our own practice, but have given them to other physicians, with the request to report to us as to their experience with it. All of them have expressed a favorable opinion of the preparations, and now commonly prescribe them. Maltine itself is palatable, and we have found it an admirable remedy in affections of the lungs and throat.

It possesses the power of producing a perfect and permanent emulsion with cod liver oil, owing to the action of the diastase present in it. With a few drops of ol. amygdal. am. added, an emulsion may be prepared, containing fifty per cent. of oil, which is pleasant to the taste, free from smell of the oil, and much more readily digested than the oil alone.

We look upon Maltine as one of the best of the preparations of malt, and the reputation of Messrs. Reed & Carnrick, as manufacturing pharmacists, is such that we have entire confidence that its present high standard of excellence will be fully maintained.

